# Recurrent Nontuberculous Mycobacterial Tenosynovitis

**DOI:** 10.31486/toj.19.0010

**Published:** 2021

**Authors:** Melissa Kwan, Richard Tupler

**Affiliations:** Department of Radiology, Ochsner Clinic Foundation, New Orleans, LA

**Keywords:** *Arthritis–infectious*, *Mycobacterium avium complex*, *Mycobacterium infections–nontuberculous*, *osteomyelitis*, *tenosynovitis*

## Abstract

**Background:** Nontuberculous mycobacteria are an uncommon pathogen for musculoskeletal infection and are difficult to treat because of delays in diagnosis, prolonged treatment requiring both antimycobacterial therapy and surgical debridement, and high rates of resistance to antimycobacterial therapy.

**Case Report:** We report the case of an 88-year-old male with recurrent *Mycobacterium avium* complex tenosynovitis despite receiving multiple courses of pharmacologic therapy and surgical debridement.

**Conclusion:** Nontuberculous mycobacterial musculoskeletal infections can be difficult to diagnose and equally difficult to treat. A combination of antimycobacterial therapy and surgical debridement is often required; however, the rate of treatment failure remains high, particularly with rapidly growing mycobacteria such as *Mycobacterium avium*.

## INTRODUCTION

Because nontuberculous mycobacterial infection is an uncommon cause for musculoskeletal infection, low clinical suspicion and an indolent clinical course make accurate diagnosis difficult. The need for an extended and multidisciplinary course of treatment complicates matters, and many patients fail treatment. We report a case of recurrent nontuberculous mycobacterial tenosynovitis in a patient with risk factors that resulted in multiple failed courses of treatment.

## CASE REPORT

**Figure 1.**
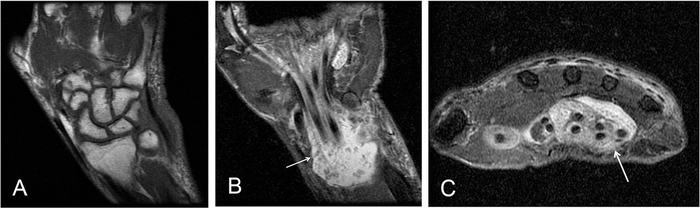
**Initial magnetic resonance imaging examination with (A) coronal T1, (B) coronal short T1 inversion recovery (STIR), and (C) axial T2 fat saturation sequences. View A shows no evidence of osseous erosions or marrow-replacing process to suggest osteomyelitis. Views B and C show fluid within the carpal tunnel and associated innumerable low T2 foci, presumed rice bodies (white arrows).**

An 88-year-old male initially presented to orthopedic surgery in January 2012 with a tender, swollen, and painful right wrist approximately 8 months after carpal tunnel release surgery performed at an outside facility. The patient reported a history of changing bird feeders and exposure to bird droppings and had no history of rheumatologic disorder. Clinical suspicion was for synovitis; however, *Mycobacterium* was also considered a possibility given the patient's history of bird exposure. The patient underwent irrigation and debridement; biopsy demonstrated thickened synovium that did not appear overtly infectious. Gram stain, acid-fast bacilli (AFB) stain, aerobic and anaerobic cultures, and fungal cultures were all negative at the time. Non-contrast–enhanced magnetic resonance imaging (MRI) (Figure 1) performed 2.5 weeks postoperatively demonstrated a large amount of fluid within the carpal tunnel, numerous low T2 signal foci suggesting rice bodies, and no osseous erosions or abnormal marrow signal to suggest osteomyelitis. The patient reported persistent wrist swelling and pain, and 1 month later, underwent repeat irrigation, debridement, and synovectomy. Gram stain, AFB stain, and cultures were again negative, and biopsy showed chronic inflammation and necrosis. Supportive care was recommended, and no antimicrobials were prescribed.

During his second postoperative course, the patient was noncompliant with the splint and reported subsequent wound dehiscence. Two weeks later, the patient underwent repeat irrigation, drainage, and biopsy, with cultures yielding *Mycobacterium avium* and *Mycobacterium intracellulare* (*Mycobacterium avium* complex [MAC]). The patient was placed on a course of clarithromycin 500 mg oral twice daily every Monday, Wednesday, and Friday (MWF), ethambutol 1,000 mg oral twice daily every MWF, and rifampin 300 mg oral twice daily every MWF. A 6- to 12-month course of treatment was planned, but the patient stopped taking the medications after 2 months because of adverse effects.

During the next 3 years, the patient presented multiple times with intermittent wrist swelling. MRIs showed similar findings to the initial study although with gradual progression of joint space narrowing, osseous erosions throughout the carpals and base of the metacarpals, and development of complex fluid at the distal radioulnar joint. No further surgical or antimycobacterial treatment was offered because of low clinical suspicion for active infection and the patient's history of noncompliance with prior pharmacologic therapy.

The patient returned in June 2015 with marked wrist swelling. Contrast-enhanced MRI showed markedly enhancing fluid with rice bodies at the distal radioulnar joint and extensive joint space narrowing, subchondral edema, osseous erosions, and synovial enhancement throughout the carpals. Fluid around the flexor tendons was decreased; however, imaging showed interval development of fluid and enhancement surrounding the extensor tendons along the dorsal aspect of the wrist, as well as a peripherally enhancing soft tissue mass surrounding the flexor carpi radialis tendon. The infectious disease physician was concerned for recurrent MAC infection, so the patient underwent tenosynovectomy of the extensor tendons and resection of the soft tissue mass adjacent to the flexor carpi radialis tendon. Pathology of the soft tissue mass showed a ganglion cyst with inflammation and thrombus, and cultures again grew MAC.

At 1.5 months after surgical debridement, the patient was started on oral azithromycin 250 mg daily and intravenous (IV) cefoxitin given his prior intolerance to oral medications; however, cefoxitin soon had to be stopped because the patient developed a rash. He was switched to IV meropenem but was on this treatment for only 3 weeks before his peripherally inserted central catheter line was removed because of surrounding skin irritation. Given the patient's preference for oral medication despite prior intolerance, he was slowly progressed to triple drug therapy with azithromycin 250 mg oral daily, rifabutin 150 mg oral twice daily, and ethambutol 400 mg oral 3 times daily, although the patient soon stopped taking ethambutol because of intolerance. He remained on dual drug therapy with azithromycin and rifabutin for 18 months.

**Figure 2.**
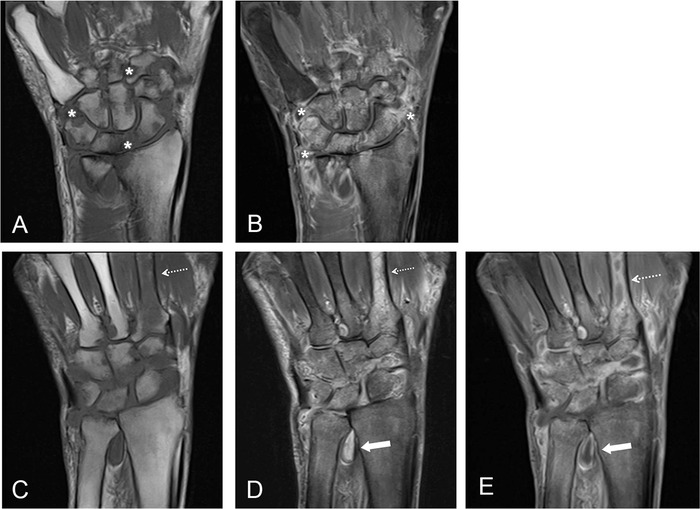
**Most recent magnetic resonance imaging examination at the carpal level with (A) coronal T1 and (B) coronal postcontrast T1 fat saturation sequences with inclusion of the metacarpals on (C) coronal T1, (D) coronal short T1 inversion recovery (STIR), and (E) coronal post-contrast T1 fat saturation sequences. Views A and B show joint space narrowing, osseous erosions (asterisks), and synovial enhancement (asterisks) throughout the carpals. Dashed white arrows show marrow edema (views C and D) and enhancement (view E) of the second metacarpal concerning for osseous involvement of the adjacent infectious process within the carpals. Additionally, complex enhancing fluid is seen at the distal radioulnar joint (solid arrows in views D and E).**

The patient did well for 1.5 years but then presented again with recurrent pain and swelling. Contrast-enhanced MRI (Figure 2) showed extensive joint space narrowing and enhancement with increased osseous erosions throughout the carpals. Complex fluid at the distal radioulnar joint and minimal fluid around the flexor tendons were seen, but marked edema and enhancement of the second metacarpal, new from the prior examination, were concerning for osseous involvement. While the other findings may be seen with inflammatory arthritis, given the presence of osseous involvement and the patient's history of MAC infection, the findings were concerning for septic arthritis and possible osteomyelitis. At the time of case report submission, the patient had undergone repeat synovectomy that showed slightly thickened tissues but no evidence of rice bodies or other evidence of infection. AFB and gram stains were negative although synovial cultures were still pending, and the patient was not reinitiated on antimycobacterial therapy.

## DISCUSSION

Nontuberculous mycobacterial musculoskeletal infections were thought to be uncommon. The incidence seems to have increased since the data from the mid-1990s, but the exact incidence is unknown as nontuberculous mycobacterial infections do not require public health reporting.^1^ Typically, mycobacterial infections present in immunocompromised hosts as a hematogenously spread disseminated disease such as vertebral osteomyelitis, whereas disease in immunocompetent hosts presents as a result of direct inoculation related to surgical procedures or penetrating injuries, the latter often contributing to osteoarticular disease via contact with objects contaminated by mycobacterial-containing soil or water.^1-3^ *M avium* in particular is primarily found in birds and the environment and is responsible for opportunistic human infections related to avian exposure.^4^ Regional degenerative soft tissue changes associated with aging may be important in allowing progression of soft tissue infection in older immunocompetent patients, most commonly during the fifth to seventh decades of life.^5^ Often, the diagnosis of mycobacterial infection within a joint or tendon is missed or delayed because of the indolent course, lack of clinical suspicion, misinterpretation of biopsies, and lack of mycobacterial cultures performed.^6-7^

The most commonly involved location for nontuberculous mycobacterial infection within the musculoskeletal system is the hand and wrist because of the relative abundance of synovium and the increased risk for inoculation through penetrating trauma.^2,6,8^ Patients usually present with swelling, drainage, or a palpable mass.^9^ Digital flexor tenosynovitis is the most common clinical presentation, with MRI showing marked synovial thickening around the flexor tendons and fluid in the tendon sheath.^6^ Early radiographic findings include subtle soft tissue swelling and juxta-articular osteoporosis, although these findings are nonspecific.^8,10^ Severe cases may progress to extensive necrosis of synovial tissue and direct extension to adjacent periosteum leading to osteomyelitis.^1,2^ The presence of rice bodies within the tendon sheaths or bursae should raise suspicion for nontuberculous mycobacterial infection.^1,3^ Differential diagnosis considerations include other infectious diseases, such as pyogenic and fungal infections, giant cell tumor of tendon sheath, pigmented villonodular synovitis, and rheumatoid arthritis, particularly given the presence of marginal osseous erosions, rice bodies, and synovial thickening and enhancement on MRI.^8^ Even at surgery, profuse and proliferative tenosynovium is commonly found and likened to rheumatoid pannus.^9^ The slow progression of joint space narrowing may help differentiate mycobacterial infection from rheumatoid arthritis and pyogenic infection, both of which demonstrate a more rapid and early course of joint space narrowing; however, the gold standard for mycobacterial infection diagnosis is tissue biopsy and synovial cultures.^1,2,6,8^

Nontuberculous mycobacterial infection should be considered in patients with chronic tenosynovitis who fail to respond to treatment and have risk factors for occupational or recreational exposure to mycobacteria, such as an aquatic or farming exposure or a history of surgical intervention.^1,2,9^ Combined surgery and antimycobacterial therapy are needed for treatment as synovectomy cannot remove all of the infected tissue but can decrease the overall disease burden, giving the pharmacologic therapy a better chance to eradicate the residual infection.^1,6,7,9^ AFB staining from synovial biopsy is often negative, with a diagnostic yield ranging from 0% to 60%, and initial histopathology evaluation may reveal granulomatous inflammation.^6,9,11^

Given the appropriate clinical history, the presence of granulomatous synovial inflammation, and negative fungal cultures, antimycobacterial therapy should be started immediately after surgery and prior to receiving definitive mycobacterial culture results, which may take several weeks.^1,5,6,9^ No consensus guidelines are currently available for specific chemotherapy for nontuberculous mycobacterial musculoskeletal infections; however, prior studies recommend clarithromycin in combination with either rifabutin, ethambutol, or ciprofloxacin, noting that toxicity may occur with interactions between clarithromycin and rifabutin.^5,6^ Prolonged therapy of at least 6 months and up to 2 years is recommended, with tailoring of pharmacologic treatment after culture results demonstrate drug sensitivities given the high rate of drug resistance among nontuberculous mycobacteria.^1,2,6,7^ However, even with thorough debridement and prolonged appropriate medical therapy, infection may recur and require repeated debridement for infection control.^1^ Treatment is more likely to fail in cases of articular infection and osteomyelitis and can lead to persistent patient disability or even amputation.^7,12^

Our patient presented with multiple risk factors for a poor prognosis despite his immunocompetency. His occupation as a farmer and his avian exposure predisposed him for infection by MAC, the most commonly identified species in bone and joint infections and known to be associated with treatment failure more than any other mycobacterial species.^7,9^ The patient's advanced age allowed for progression of disease. If the patient's initial AFB stains and culture had returned positive for mycobacteria, he could have been started on pharmacologic treatment at least 6 weeks earlier than he was. However, clinical suspicion for mycobacterial infection was low at the time, and the patient's exposure to birds was a retrospective discovery.

The indolent course and delayed diagnosis were compounded by noncompliance with postoperative management and inadequate pharmacologic therapy, attributable to both noncompliance and patient intolerance. Although no consensus guidelines for pharmacologic therapy of nontuberculous mycobacterial musculoskeletal infections are available, the patient's initial course of pharmacologic treatment was likely inadequate because of its 2-month duration; despite apparent improvement in clinical symptoms, most recommendations call for a minimum of 6 months of pharmacologic treatment.^1,2,6,7^ Given the patient's most recent MRI findings showing probable osseous involvement of the second metacarpal, his second course of treatment in 2015 was also likely inadequate despite the 18-month duration because of the delay in initiation of antimycobacterial therapy following surgery and the treatment regimen containing only 2 of the recommended 3 medications. A likely prognosis is that our patient will continue to fail treatment given that more severe infection with articular involvement and osteomyelitis as suspected on his most recent imaging has been shown to lead to treatment failure more commonly than tenosynovial infection alone.^7^

## CONCLUSION

Clinical suspicion for nontuberculous mycobacterial infection should be increased for patients with a history of surgery or an aquatic or farming exposure, chronic tenosynovitis, radiologic findings of rice bodies within tendon sheaths and bursae, and histopathologic findings of a granulomatous synovial infection. High clinical suspicion and early initiation of treatment following surgical debridement will increase the likelihood of successful treatment. Delayed diagnosis and treatment will likely lead to treatment failure with recurrent and progressive infection that may result in significant patient morbidity.

## Notes

This article meets the Accreditation Council for Graduate Medical Education and the American Board of Medical Specialties Maintenance of Certification competencies for Patient Care and Medical Knowledge.
